# What happens to brain outside the thermal ablation zones? An assessment of needle-based therapeutic ultrasound in survival swine

**DOI:** 10.1080/02656736.2022.2126901

**Published:** 2022

**Authors:** Benjamin Szewczyk, Matthew Tarasek, Zahabiya Campwala, Rachel Trowbridge, Zhanyue Zhao, Phillip M. Johansen, Zachary Olmsted, Chitresh Bhushan, Eric Fiveland, Goutam Ghoshal, Tamas Heffter, Farid Tavakkolmoghaddam, Charles Bales, Yang Wang, Dhruv Kool Rajamani, Katie Gandomi, Christopher Nycz, Erin Jeannotte, Shweta Mane, Julia Nalwalk, E. Clif Burdette, Gregory Fischer, Desmond Yeo, Jiang Qian, Julie Pilitsis

**Affiliations:** a Department of Neurosurgery, Albany Medical Center, Albany, NY, USA; b Robotics Engineering Department, Worcester Polytechnic Institute, Worcester, MA, USA; c GE Global Research Center, Niskayuna, NY, USA; d Department of Neuroscience and Experimental Therapeutics, Albany Medical Center, Albany, NY, USA; e Charles E. Schmidt College of Medicine, Florida Atlantic University, Boca Raton, FL, USA; f Acoustic MedSystems, Inc, Savoy, IL, USA; g Animal Resources Facility, Albany Medical Center, Albany, NY, USA

**Keywords:** Brain metastases, focused ultrasound, magnetic resonance-guided robotically assisted delivery, needle-based therapeutic ultrasound, histological analysis

## Abstract

**Background:**

In stereotactic radiosurgery, isodose lines must be considered to determine how surrounding tissue is affected. In thermal ablative therapy, such as laser interstitial thermal therapy (LITT), transcranial MR-guided focused ultrasound (tcMRgFUS), and needle-based therapeutic ultrasound (NBTU), how the surrounding area is affected has not been well studied.

**Objective:**

We aimed to quantify the transition zone surrounding the ablation core created by magnetic resonance-guided robotically-assisted (MRgRA) delivery of NBTU using multi-slice volumetric 2-D magnetic resonance thermal imaging (MRTI) and subsequent characterization of the resultant tissue damage using histopathologic analysis.

**Methods:**

Four swine underwent MRgRA NBTU using varying duration and wattage for treatment delivery. Serial MRI images were obtained, and the most representative were overlaid with isodose lines and compared to brain tissue acquired postmortem which underwent histopathologic analysis. These results were also compared to predicted volumes using a finite element analysis model. Contralateral brain tissue was used for control data.

**Results:**

Intraoperative MRTI thermal isodose contours were characterized and comprehensively mapped to post-operative MRI images and qualitatively compared with histological tissue sections postmortem. NBTU 360° ablations induced smaller lesion volumes (33.19 mm^3^; 120 s, 3 W; 30.05 mm^3^, 180 s, 4 W) versus 180° ablations (77.20 mm^3^, 120 s, 3 W; 109.29 mm^3^; 180 s; 4 W). MRTI/MRI overlay demonstrated the lesion within the proximal isodose lines. The ablation-zone was characterized by dense macrophage infiltration and glial/neuronal loss as demonstrated by glial fibrillary acidic protein (GFAP) and neurofilament (NF) absence and avid CD163 staining. The transition-zone between lesion and normal brain demonstrated decreased macrophage infiltration and measured ~345 microns (*n* – 3). We did not detect overt hemorrhages or signs of edema in the adjacent spared tissue.

**Conclusion:**

We successfully performed MRgRA NBTU ablation in swine and demonstrated minimal histologic changes extended past the ablation-zone. The lesion was characterized by macrophage infiltration and glial/neuronal loss which decreased through the transition-zone.

## Introduction

Thermal ablation techniques are being explored as minimally- or noninvasive alternatives to surgery for primary and metastatic brain tumors [1]. Magnetic resonance-guided (MRg) laser interstitial thermal therapy (LITT) has experimentally been used to ablate radioresistant brain metastases [2,3], and transcranial MR-imaging guided focused ultrasound (tcMRgFUS) has experimentally been used in the treatment of glioblastoma multiforme (GBM) patients [1,4]. Interstitial needle-based therapeutic ultrasound (NBTU) delivers precise ablative therapy utilizing a similar clinical workflow to LITT. NBTU has been shown preclinically to directly deliver lower doses of acoustic energy more efficiently to the target tissue while sparing the surrounding regions [1,5]. We therefore posit that NBTU has greater potential to treat inaccessible tumors near to or involving vasculature [6,7]. Further, NBTU does not suffer from the limitations of tcMRgFUS to ablate brain metastases at the white-gray matter junction because of calvarial acoustic attenuation.

The device which provides NBTU consists of longitudinally stacked piezoelectric transducers which allow for the creation multi-angular beam patterns [7,8]. Modeling suggests that similarly to radiotherapy, remaining acoustic energy is distributed within the transition zone between the focal spot and the surrounding normal tissue and can be characterized by measuring the distance between specific isodose lines within a dose gradient [7,9,10]. Characterizing the transition zone at different dosage and delivery patterns will allow for optimal NBTU treatment parameters for specific conformal targets with minimal collateral damage to the surrounding tissue.

Although previous modeling and *ex vivo* studies have shown that isodose lines provide insight into treatment zones in NBTU, no previous study has characterized the transition zone within *in vivo* brain tissue. In order to translate NBTU to human clinical trials and therapy, characterization of this transition zone is paramount. Here we mapped intraoperative MRTI thermal isodose contours of NBTU with post-operative MRI images and analyzed histological tissue sections in four survival swine.

## Materials and methods

This project was approved by the Albany Medical College (AMC) Institutional Animal Care and Use Committee (IACUC). MRgRA delivery of NBTU was conducted on four female Yorkshire swine (6–8 weeks old) weighing between 11.8 kg and 20 kg. MRI was used to serially monitor the ablation zone and the surrounding area at 2 weeks and 4 weeks post-NBTU therapy. Neurological assessments were performed before treatment and iteratively after for 4 weeks. Each survival animal was euthanized without recovery from anesthesia after the 4-weeks MRI. Brain collection was performed immediately following euthanasia.

### Surgery

Details of the procedure, robot, and ablation have been previously published [1,3,11,12]. Under anesthesia, the surgical site was prepped and draped using aseptic techniques. A frontal incision was made using a #10 blade, and electrocautery was employed to further dissect to the calvarium. A 1 cm-wide burr hole was created using a Midas Rex high-speed drill (Medtronic, Dublin, Ireland). The dura was opened with a #11 blade and a corticotomy was performed and hemostasis obtained. The incision was irrigated, and small pieces of Surgifoam (Ethicon, Somerville, NJ) were placed within the burr hole. Dermalon sutures (Covidien, Medronic, Dublin, Ireland) were used to close the skin. The pig was transferred to the MRI suite under continuous monitoring and secured in position. Care was taken to ensure that the burr hole was positioned in-line with the robot arm, which was secured onto the bed of the MRI scanner adjacent to the swine.

The MRg robotic assistant was designed to act as an actuated MRI-compatible stereotactic frame. Planning was performed by identifying target and skull entry positions on the intraoperative MR images. The swine heads were registered to the MRI space using an MRI-specific fiducial Z-frame (similar to those used in deep brain stimulation) on which the swine head sits. The MRI-specific registration plate has three sides containing fluid-filled bars (MR-SPOT^®^ Skin Markers, Beekley Medical, Bristol, CT) which appear hyper-intense on MRI and have a specific orientation (vertical on two ends and diagonal in the middle). The exact axial location along the frame can be determined by assessing the location of the diagonal bar to either end. Coordinates of entry were determined using volumetric 3 D T1-weighted (T1w) BRAVO (BRAin VOlume) MR images (echo time (TE) 3.3 ms, repetition time (TR) 8500 ms, flip angle (flip) 12, inversion time (TI) 450 ms, 22 cm field of view (FOV), 256 × 256 matrix, 1.6 mm slice thickness). The TheraVision software (Acoustic MedSystems, Inc., Savoy, IL) controlled the movement of the robot based on the received MR input. The robot was registered to the scanner coordinates and the targeting information sent to it. The robot aligned the needle-based therapeutic ultrasound probe and inserted it along the intended trajectory. The pose of the robot, and that of the probe that is held in its end effector, is used to identify the scan plane geometry (perpendicular to the probe) to use for the MR thermometry imaging that was used to monitor the delivery of ablation.

Once coordinates were established, the surgeon exposed the corticotomy and MRgRA delivery of a cannula into the brain, at the pre-determined position and depth, was performed. The MR-compatible NBTU applicator (Acoustic MedSystems, Inc., Savoy, IL) was inserted within the cannula to the correct target region. Along the longitudinal axis of the NBTU applicator, transducers allowed MRgRA delivery of NBTU at radial 180° or 360° insonation patterns in the cerebral tissue. A volumetric MRTI algorithm was established to track live changes in thermal dose within multiple 3- or 5-mm contiguous slices. MRTI was achieved using a spoiled gradient echo sequence (SPGR) with TE 13 ms, TR 90 ms, flip 30, 19 cm FOV and 256 × 256 matrix. Corrected proton resonance frequency shift (PRFS) maps were computed as:

$$\Delta T(x,y)=\frac{\Delta \phi_{w}(x,y)-\Delta \phi_{cor}(x,y)}{\gamma \alpha B_{0}TE}$$

where $\phi_{cor}(x,y)$ represents the image phase in a non-heated region of the brain. $\Delta \phi_{w}(x,y)$ represents the water-based phase difference maps corrected by the interpolated phase difference maps of the $\Delta \phi_{\text{cor}}(x,y)$. Here, the PRFS thermal coefficient for water-based tissues (α) was not experimentally calibrated [13]. Hence, a nominal value of a=−0.01ppm/°C was chosen.

MRTI slices were aligned to create a thermal dose map, which was overlayed with the T1w BRAVO MRI images. Following ablation, T1w BRAVO, Fast Spin Echo (FSE) T2-weighted (TE 40 ms, TR 2500 ms, ETL 4, flip 125, 19 cm FOV, 384 × 256 matrix), and diffusion-weighted imaging (DWI) (TE 90 ms, TR 3500 ms, flip 90, 16 cm FOV, 144 × 90 matrix, *B*-value = 1000) MRI sequences were run. Apparent diffusion coefficient (ADC) maps were subsequently computed from DWI MRI data. After post-ablation MRI scanning, the probe and cannula were withdrawn from the tissue and the incision closed. The animal was extubated and supervised throughout the duration of its recovery.

Animals were closely monitored by the surgical team for the 24 h post-surgery.

Following surgery, daily neurological and physical assessments were performed for the seven days following NBTU, and weekly thereafter. Exams included a scaled assessment of vocalizations, appearance, agitation, aggression, and social behavior. Physical exams assessed breathing, neurological signs, motor weakness, and skin condition.

### MRI analysis

T1w with and without contrast (T1w + c; gadobutrol 1 mmol/mL), T2w, and DWI MRI images were also acquired 2 weeks and one month after NBTU therapy. The same intubation and anesthesia protocol was followed for post-op MRI scans as detailed above for the initial surgery. After the second MRI, animals were euthanized. The brains were dissected and immersed in paraformaldehyde (PFA, 4%). During post-processing, the post-ablation and two post-operative T1w BRAVO scans were registered to the pre-ablation T1w BRAVO imaging. MRTI isodose lines were overlaid over images at all four timepoints (pre-ablation, immediately post-ablation, 2 weeks post-ablation, and 4 weeks post-ablation) to analyze change over time within the ablation area and surrounding tissue.

**Figure 1.**
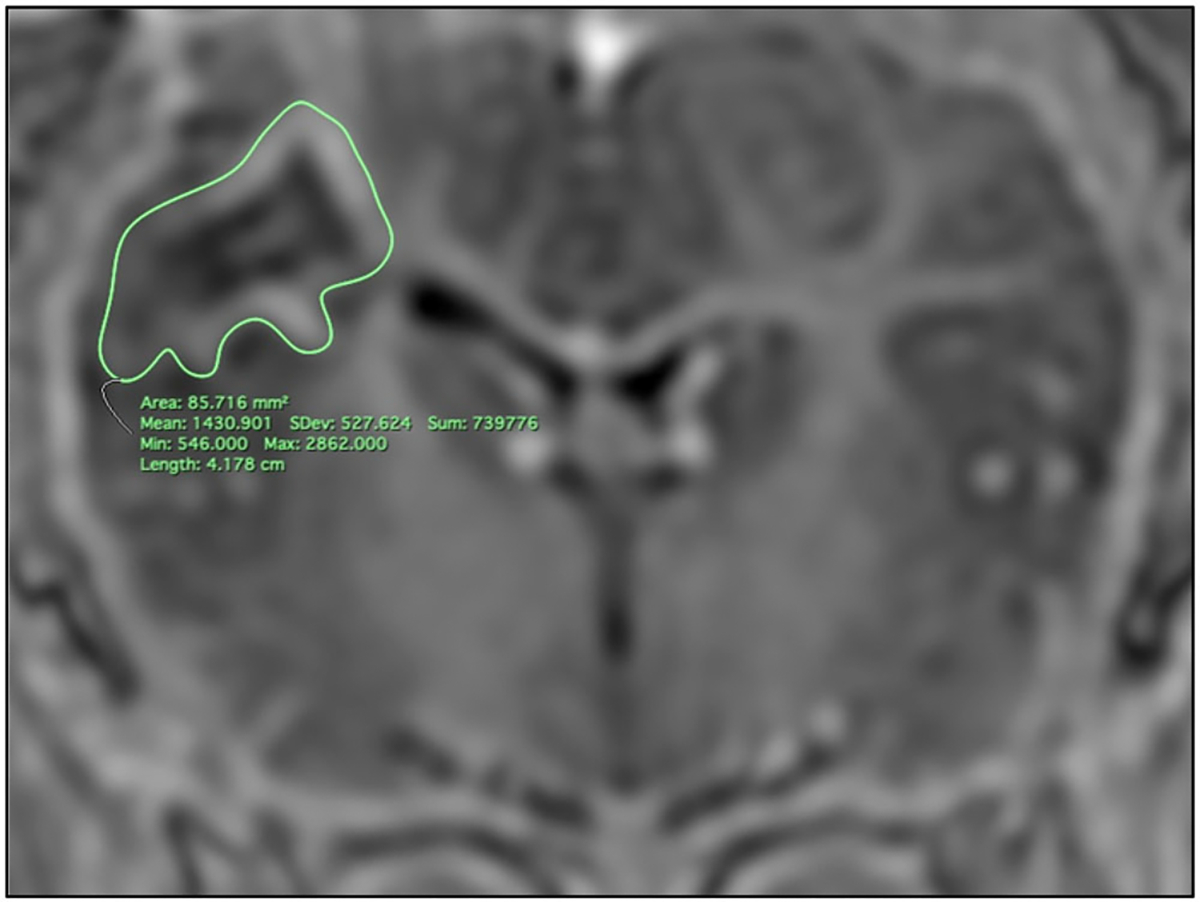
Coronal T1-weighted MRI with contrast 2 weeks post-ablation demonstrating the method for measuring the ablative lesion created by the 180° probe (4 W, 180 s) with the probe facing anteriorly. The lesion is seen here circled in green and appears as an area of hypo-intensity with a rim of surrounding contrast enhancement.

Thin-cut (4 mm) T1w BRAVO images with and without contrast were re-formatted and viewed in multiple imaging planes. The areas of MRI which demonstrated changes (typically signal hyperintensity with central hypointensity) were measured using open-source Horos medical DICOM viewer with the closed polygon region of interest (ROI) selection tool (Figure 1). Ablation volumes were calculated by summating the areas demonstrating change per slice multiplied by the slice thickness.

### Histological analysis

Swine brain tissue was acquired 4 weeks post-ablation and remained emerged in PFA in a 4 °C refrigerator for approximately one week before slicing. The brain was aligned within the Zivic Pig Brain Slicer (Zivic Instruments, Pittsburgh, PA) and 5 mm coronal slices were made from the frontal to occipital poles. We focused on slices where the ablation or probe track could be visibly detected, and these slices were halved to result in 2.5 mm slice thickness for paraffin embedding. The paraffin blocks were sectioned at 5 *μ*m using a Microm HM325 microtome (Thermo Scientific). The sections were processed per routine histology protocol, and then stained for H&E, GFAP (Cell Marque, Rocklin, CA; 1 *μ*g/mL), NF (Cell Marque, Rocklin, CA; 0.09 *μ*g/mL), and CD163 (Novus Biologicals, Littleton, CO; 1:400).

The H&E, NF, GFAP, and CD163 stained slides were examined, and the ablation and transitional zones were identified, characterized, and outlined. Ablation zones were calculated using the following formula for each individual swine: *V* (volume) of lesion = *A* (area) × Thickness of brain slices (fixed at 2.5 mm). Each slice of interest was divided into left and right hemispheres and the contralateral side used as a control.

**Figure 2.**
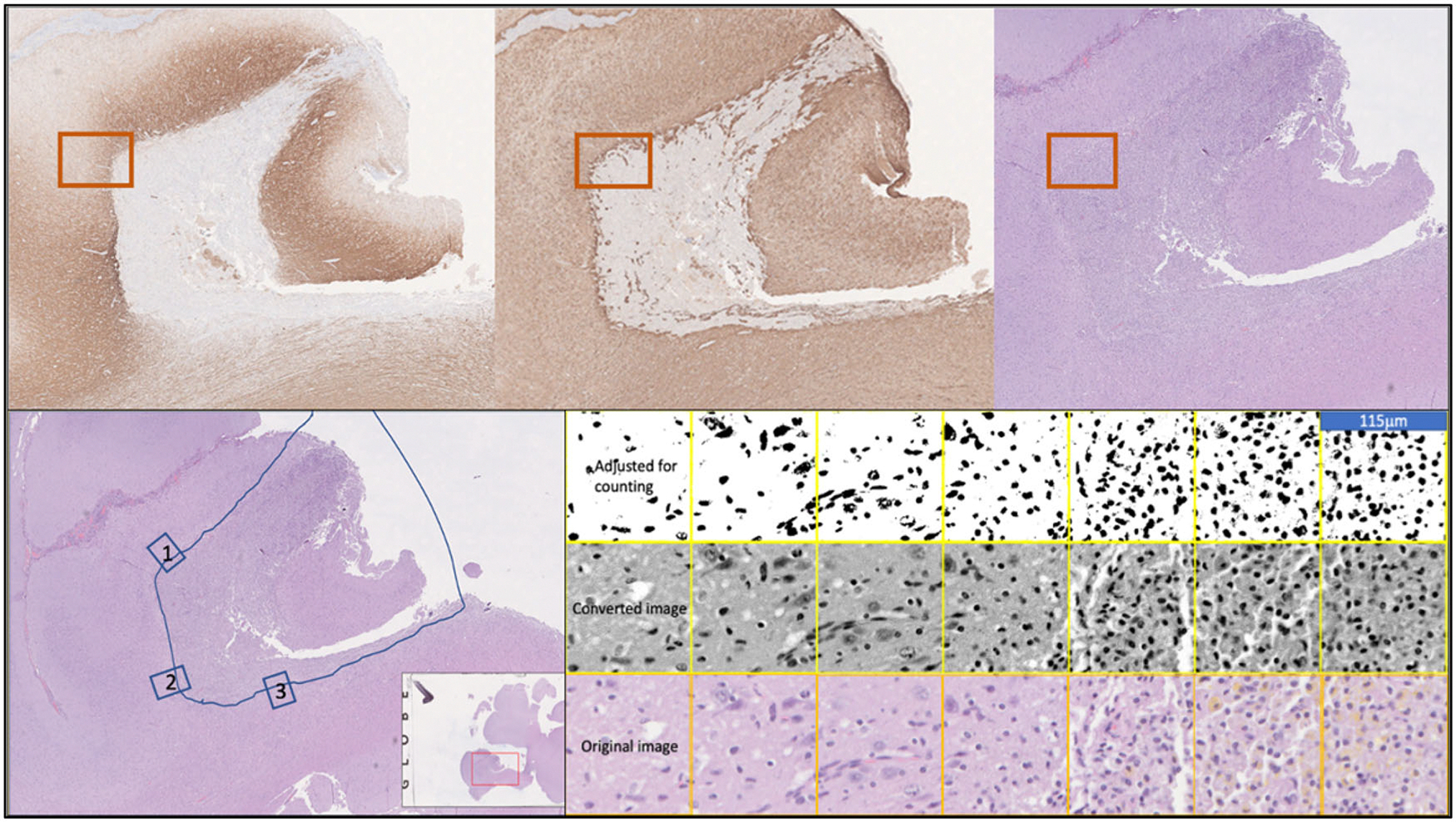
Transect method for cell counting across the boundary of the lesion (bottom left/right); (top, left: GFAP; top, middle: NF; top, right: H&E).

**Figure 3.**
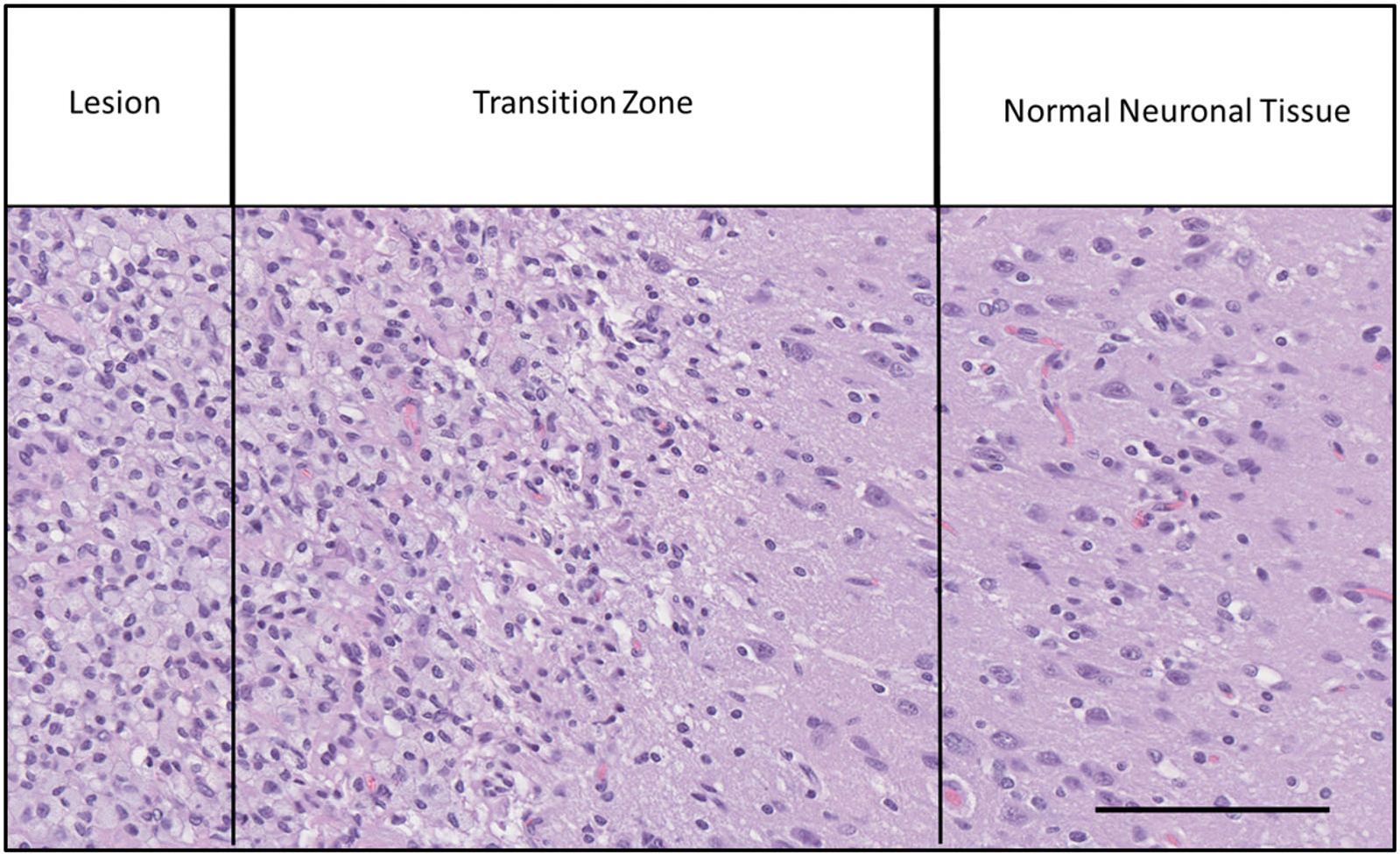
Representative sample of macrophage infiltration at the lesion margin. Lesion, with foamy macrophages, shown here on the left; transition zone in the center; normal neuronal tissue, for this brain region, shown on the right (40×, black scalebar right, 115 μm).

Techniques were employed to analyze cellular density and macrophage activation in surrounding brain tissue. Cell counts were done as a transect across the defined transition zone (total distance 690 um, divided across six, 115um ‘blocks’) (Figure 2). ImageJ (Wayne Rasband and contributors, National Institutes of Health, USA) [14] was used to image the transect zone from −115 microns (foamy macrophages) to 575 microns (normal tissue) – equaling a total of six blocks. Three samples along the lesion site (*n* = 3) were used for each animal. We converted each image for black/white thresholding. Next, we used imageJ to count the total number of cells in each block [14]. For analysis, we used Graphpad Prism (GraphPad Software, San Diego, CA) to plot our data and made a one-way ANOVA on the cell counts across the transition zone in each image. Figure 3 demonstrates the transition from lesion to normal brain tissue at a moderate magnification (40×).

### MRTI analysis

MRI images which had demonstrated obvious changes from different imaging sessions were co-registered together using a custom pipeline that uses a grid search-based initialization to achieve robust alignment even in the presence of large transformation [15]. We calculated the transformation between images from the same session as a rigid transformation and that between different sessions as an affine transformation. Further, as there was sizable growth of the subjects over 4-weeks period, we used brain masks to limit computation of our normalized mutual-information based cost function. After co-registration, all images were resampled with isotropic voxel size of 0.4 mm^3^ in a single-step interpolation to avoid image blurring. The custom pipeline is implemented in Elastix framework [16] and co-registered images can be viewed in 3 D Slicer (http://www.slicer.org) or similar visualization tools. The thermal isodose maps corresponded to 20, 50, 100, 200, 500, 1000, and 2000 CEM43. The 2-week T1w with contrast images were chosen because these provided the best visualization of the lesion. The immediate post-ablation images tended to have hyperintensity in excess of the lesioned area, which may represent a transient post-ablation effect or probe artifact. This phenomenon was not observed after probe removal in later experiments. MRTI ablation volumes were also calculated at the 70 CEM43 threshold as previously described by our group [17] and compared to a predicted finite element model (FEM) and histologic volumes.

### Finite element model analysis of NBTU in brain tissue

The FEM for simulating thermal propagation and CEM43 threshold calculation were informed by NBTU parameters during surgery, while the parameters for the application were based on liver studies and phantom simulations [18,19]. We enhanced our existing numerical models with an acoustic medium based on brain-tissue specific parameters for comparison to our *in vivo* animal data [20,21].

The FEM model was implemented in COMSOL Multiphysics 5.6 (COMSOL AB, Stockholm, Sweden) to model the probe’s mechanical deformation, resultant applicator stationary acoustic pressure field, and the thermal dose threshold of 70 CEM43. A cross-sectional model of the probe’s geometry was created, surrounded by a 100 mm × 100 mm (L × W) 2 D acoustic medium. The probe material parameters were reported previously [19], and the brain tissue referenced acoustic medium material properties are described in Table 1. Far-field and thermally insulated boundary conditions were applied to the acoustic medium, and no reflection of ultrasonic waves from edges was assumed. A frequency domain study was conducted to obtain the acoustic pressure field produced by the applicator, applying a tetrahydral mesh with a maximum element size of λ/6, where λ represents the wavelength of the produced ultrasonic wave within the medium.

A bioheat transfer time domain study was then conducted to validate the heat deposition by the simulated acoustic pressure field. Base on Pennes’ equation [23]:

$$\rho c_{t}\frac{\partial T}{\partial t}=\nabla \cdot k\nabla T-\rho_{b}\omega_{b}c_{b}\left(T-T_{b}\right)+Q_{p}+Q_{m}$$

where ρ (kg/m^3^) is the tissue density, $c_{t}$ (J/kg/°C) is the tissue specific heat capacity, k (W/m/°C) is the tissue thermal conductivity, T (°C) is the current temperature, $T_{b}$ (°C) is the temperature of blood, and we assumed the initial temperature of both tissue and blood as 37 °C. $\rho_{b}$ (kg/m^3^) is the blood density, $\omega_{b}$ (kg/m^3^/s) is the blood perfusion, $c_{b}$ (J/kg/°C) is the blood specific heat capacity, and $Q_{p}$ (W/m^3^) is the heat deposition due to sonication given by:

$$Q_{p}=\frac{\alpha_{\mathrm{atten}}p^{2}}{\rho c}$$

where $\alpha_{\mathrm{atten}}$ (Np/m) is the medium attenuation, p (Pa) is the acoustic pressure field, ρ (kg/m^3^) is the medium density, c (m/s) is the speed of sound within the medium. $Q_{m}$ (W/m^3^) is metabolic heat, which is the heat generated by the tissue [24]. Predicted thermal dose maps for NBTU probes showed inhomogeneous CEM 43 thermal dose patterns because of the random blood perfusion and metabolic heat influence generated by COMSOL Multiphysics (COMSOL AB, Stockholm, Sweden).

**Figure 4.**
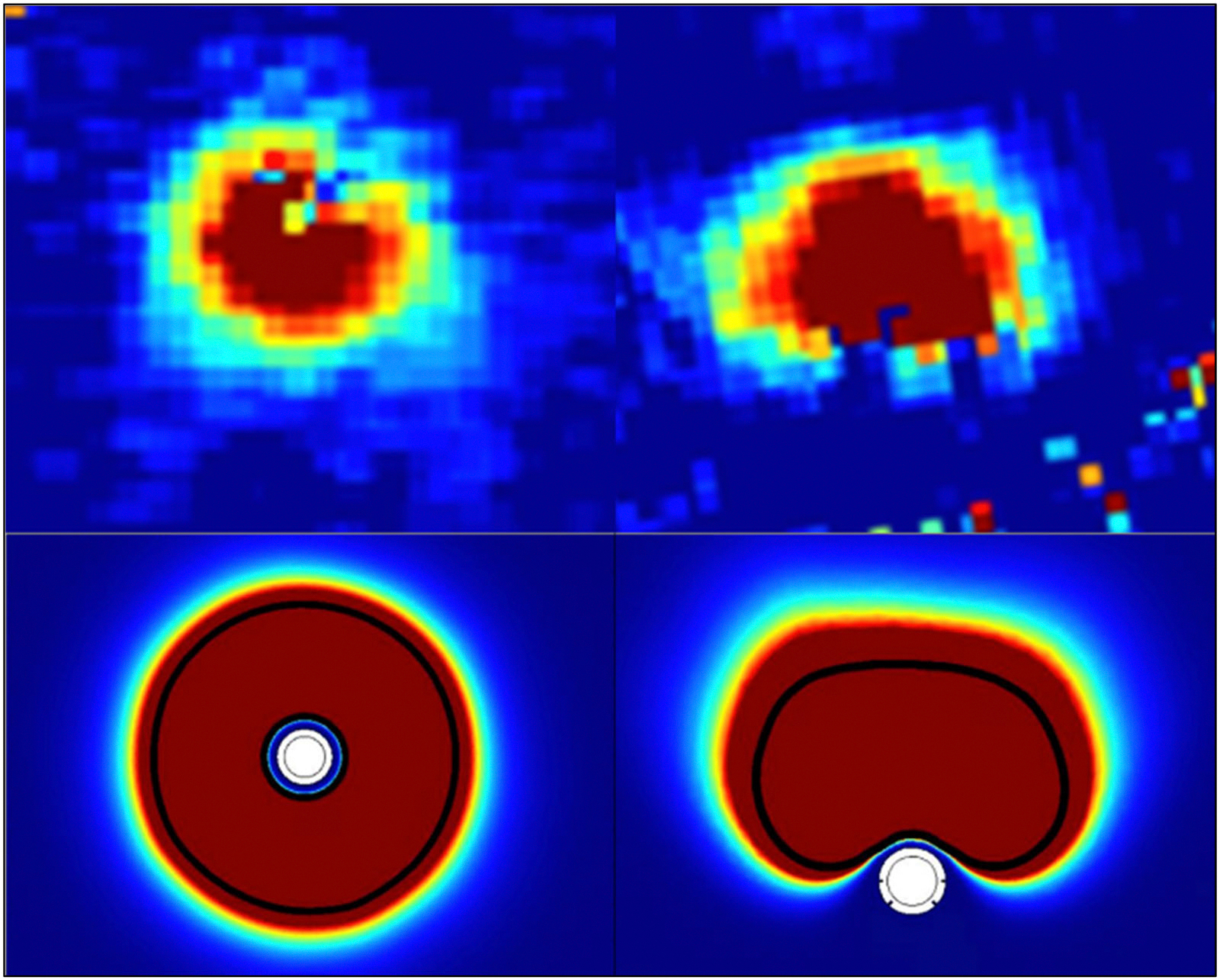
Demonstration of lesions formed with NBTU probes via real-time MRTI and FEM. Top left – live MRTI thermal map demonstrating 360° lesion at time of maximal temperature (3 W, 120 s). Top right – live MRTI thermal dose map demonstrating 180° lesion (3 W, 180 s). Bottom left – FEM of the 70 CEM43 thermal dose map for the 360° probe with ablation parameters of 4 W, 120 s in middle section of transducer. Bottom right – demonstration of the 70 CEM43 thermal dose map for the 180° probe with ablation parameters of 4 W, 180 s in the middle section of the transducer. In both instances, the solid black line represents the 70 CEM43 isodose line.

This thermal simulation used a mesh of tetrahydrals with a maximum element size of 1 mm. A general form partial differential equation (PDE) study was then conducted to calculate the CEM43 thermal dose map and 70 CEM43 isodose line (Figure 4). Using the same volume acquisition method for MRT images, we used surface integration to calculate the 70 CEM43 isodose line surrounded area of transducer in the middle section. Areas of additional slides with 5 mm slice thickness were acquired, multiplied by 5 mm, and summed together to calculate the numerical predicted ablated volume.

## Results

Four swine underwent a total of four frontal MRgRA NBTU lesions and were observed for 4 weeks prior to euthanasia. The average age at time of the ablation was 7 weeks and average weight was 19.53 ± 0.98 kg. The average age and weight of the swine at time of sacrifice was 11.79 weeks and 35.68 ± 1.56 kg (83% increase), respectively. The ablation doses and duration are listed in Table 2. The mean ± SEM absolute maximal temperature increase during the ablation was 60.5 ± 1.32 °C. The mean ± SEM time for the temperature to return to baseline was 135 ± 6.46 s. None of the experimental swine suffered from any long-term neurologic or behavioral complications. One swine took longer to wake up from general anesthesia and was noted to have discomfort/weakness in the hind legs on post-operative day 1, which resolved within a week, as well as a post-operative fever which was treated with acetaminophen.

**Figure 5.**
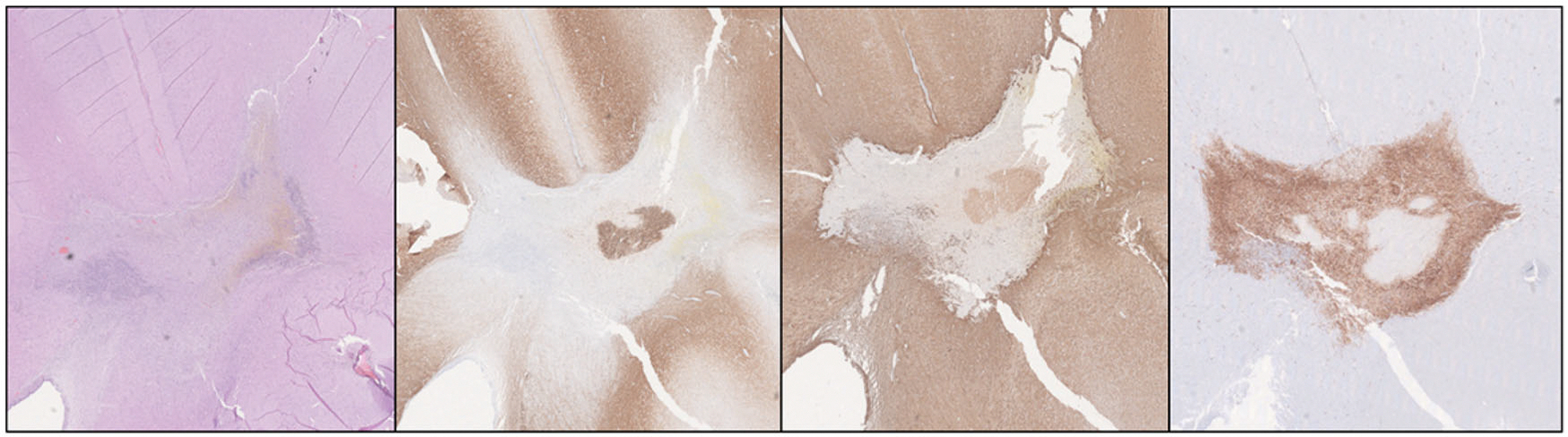
Demonstration of clear boundary of lesion on histologic staining of lesion created by 180° probe at 3 W, 180 s (first: H&E; second: NF; third: GFAP; fourth: CD163) demonstrating loss of GFAP/NF and increased CD163 staining, consistent with neuronal/glial loss and infiltration by macrophages.

**Figure 6.**
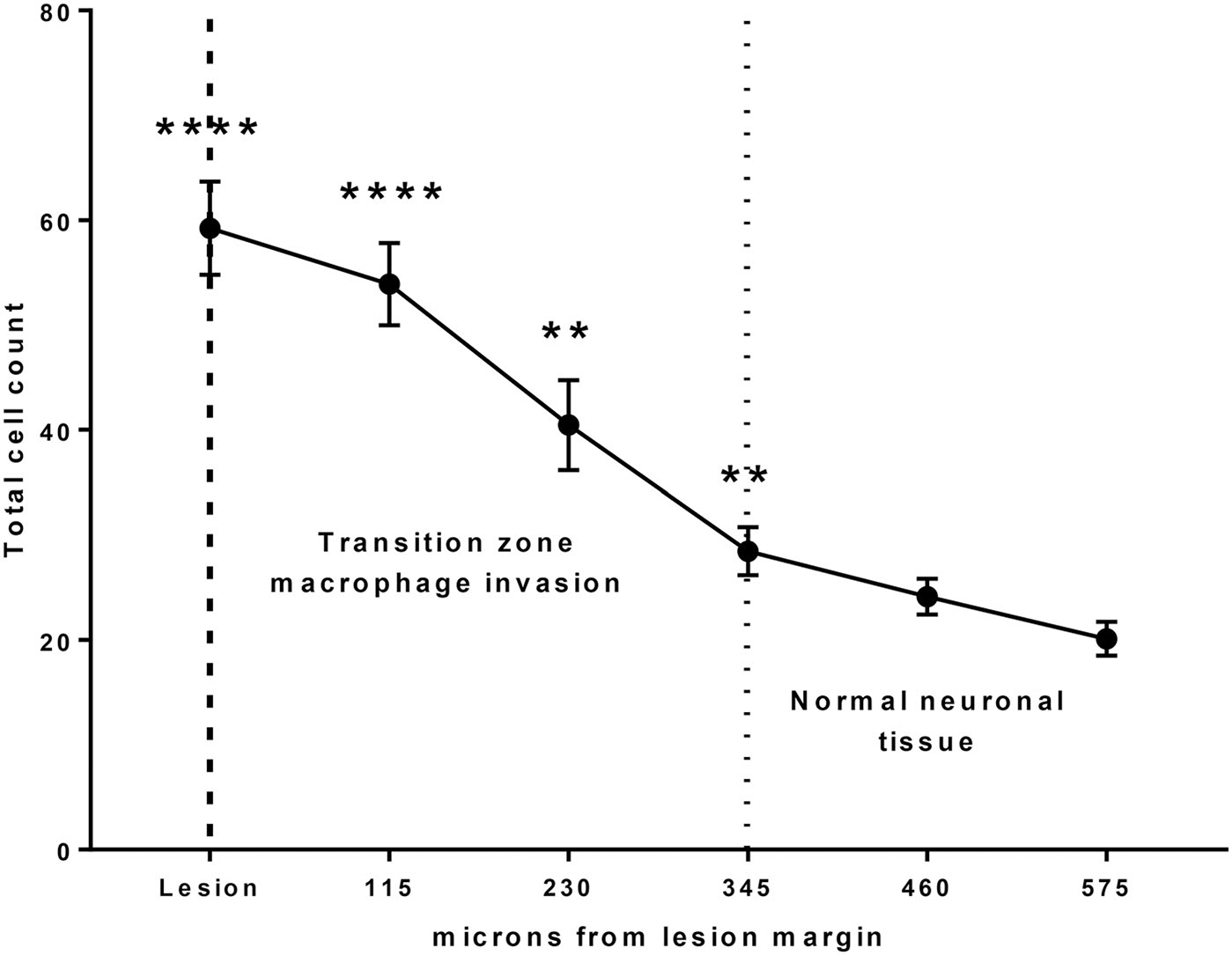
Quantification of transition zone based upon cell-counting transect method. There is a significant increase in cell count 345 microns beyond the lesion margin and into the surrounding neuronal tissue. After 345 microns out, tissue appears to be normal. **, **** = *p* < .05 and *p* < 0.001, respectively, compared to cell count at 575 microns from lesion.

Histological characterization of the lesion post-treatment by H&E, GFAP, NF, and CD 163 staining showed characteristics of macrophage infiltration in the lesional zone, a transition zone, and normal tissue. The transition zone was demarcated by an increase in cell density and macrophage activation. Cell counts were done as a transect across the defined transition zone. The transect technique could not be applied to the histologic sectioning in the third swine due to tissue destruction and fragmentation during the fixation and harvesting process. The lesions have swollen cells that are considered ‘foamy macrophages’ for about 115 microns. The transition zone was 345 microns on average across the samples (Figures 5 and 6).

### MRI, histology, and FEM correlations

**Figure 7.**
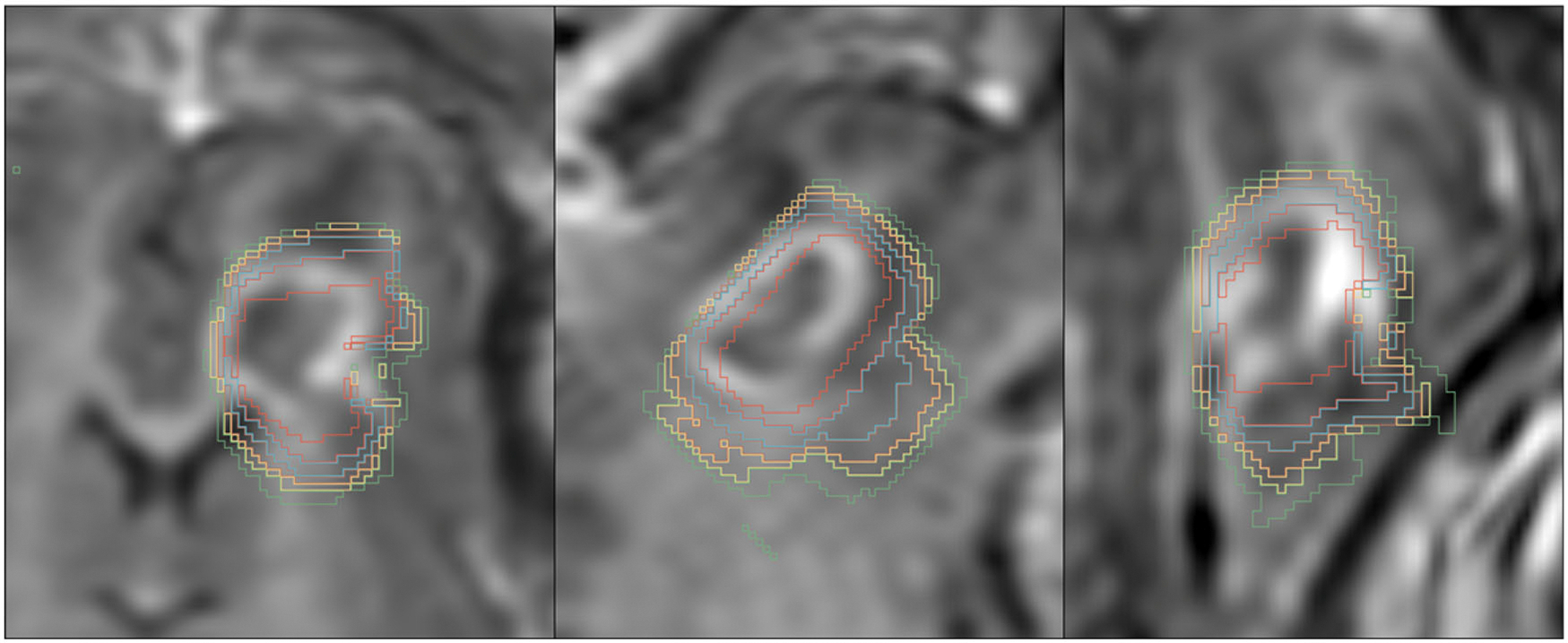
Axial (left), sagittal (middle), and coronal (right) T1-weighted MRI with contrast 2 weeks post-ablation with the 180° probe (3 W, 180 s). The colored zones correspond to 20, 50, 100, 200, 500, and 1000 CEM43 approaching the center of the lesion. These demonstrate that the intermediate- term MRI changes (2 weeks) of the ablation area are in the approximate area of the proximal intraoperative isotherms.

MRI ablation volumes were calculated by summating the areas demonstrating change per slice multiplied by the slice thickness. Initial MRTI isodose lines corresponding to 20, 50, 100, 200, and 500 CEM43 were overlain and demonstrated good correlation with the lesion boundary in 3 imaging planes (Figure 7). Ablation regions became progressively less apparent over time (Table 3) with volumes described by an exponential curve with a half-life of 5.19 days (*R*^2^ = 0.9921). There was no significant difference between the observable ablation volumes on the 4-week MRI and histologic volumes (*p* = 0.347). The MR volumes at 4 weeks were significantly less than the MRTI ablation volume measurements (*p* < 0.001; Table 2). There was no significant interrater variability on MR volume measurements (*p* = 0.954 with limits of agreement between P1 and P2 measurements ranging from −0.309 cm^3^ to 0.255 cm^3^ with a mean ± SEM difference of −0.027 ± 0.030 cm^3^). There were no significant differences between the experimental MRTI volumes and the predicted FEA analysis (*p* = 0.508; limits of agreement ranged from −0.146 to 0.001 cm^3^ with a mean difference of −0.073 ± 0.019 cm3 (6.70%).

## Discussion

### NBTU as a hyperthermic modality

LITT is now very commonplace for multiple indications including epilepsy, tumors, and radiation necrosis. This technology relies on the transfer of energy through high intensity light generated by a laser; therefore, the energy transfer occurs directly at the point where the light contacts the tissue. Alternatively, NBTU uses high intensity ultrasound and generates heat through mechanical ultrasonic heat transfer and is an efficient approach to deliver energy to induce hyperthermic cell coagulative necrosis by protein denaturation and DNA damage when temperature increases above ~55–60 °C [25–27]. Additionally, in this way the heat transfer can occur beyond boundary of the probe. Absorption of acoustic energy is proportional to frequency and is also dependent on tissue-specific properties such as the acoustic absorption coefficient [28,29]. This may be advantageous because of a lower theoretical risk of charring inhibiting heat transfer; indeed, Djin et al. have demonstrated that heating pattern is not affected after repeat sonications after a slight delay [30]. Another advantage of NBTU may be the ability to create complex geometries with less manipulation of the catheter. The lesional geometry for tcMRgFUS is limited to ovoid. The geometry for LITT was historically limited to confocal, however now with side-firing LITT variable complex geometries are possible. Here we have demonstrated the ability to create 180° NBTU ablations and have validated the technology for narrow-beam and very-narrow beam probe configurations (90°, 60° currently in development). Moreover, because the NBTU probes can have multiple elements with multiple sections corresponding to different channels, it will be possible to create different ablation patterns in one setting without having to change the probe. This could increase the efficiency of the workflow for a treatment session. Therefore, there are several reasons that NBTU may be an efficient way of performing thermal ablation with theoretical advantages over existing thermal therapies. In addition, there is evidence that ultrasound energy is less likely to damage blood vessels and could be used with vascular lesions, where LITT is contraindicated.

The workflow between LITT and NBTU is very similar, including the ability to monitor both using MRTI. Comparable efficacy and safety profiles have been demonstrated with MRTI use across disciplines, including use of tcMRgFUS and LITT, in the treatment of thalamic lesions and recurrent glioblastomas, and studies in both rabbit and swine models [17]. The main difference in this case is that we have proposed MR-guided robotically-assisted NBTU, where the targeting, probe insertion/localization, and ablation can all occur sequentially in one treatment setting. With LITT, the catheter is often placed using well-established stereotactic techniques (stereotactic frames, conventional robots, 3D plastic frames) based on pre-operative imaging; therefore, it is unknown where the probe is until the patient has moved to the MRI scanner. MRgRA NBTU would prevent aberrant probe placement since the probe is placed in real time under MRI-guidance. This may help to optimize probe placement (and therefore treatment) since it is under direct MR visualization.

Our histological findings show a sharp boundary between ablation and the transition zone. The main notable change in the transition zone was an increase in macrophages. Other studies on MRI findings in NBTU have demonstrated immediate changes on T1w + c and T2 changes appear approximately 2 h after treatment [30].

### Comparison to histologic/radiologic findings in other hyperthermic modalities and SRS

Thermal ablative therapies are becoming increasingly used for treatment of tumors as well as movement disorders [31–33]. LITT has been investigated in a cat model with description of histologic and pathologic findings after intracranial laser photocoagulation of normal brain tissue [34]. At 2 days, the MRI findings on MRI suggested a necrotic thermal lesion with a central cavity surrounded by concentric zones of dense and dispersed coagulative necrosis. This appeared to become less obvious during the 14-day follow-up study. The best approximation of the lesion in this case was on the 2 day T1 + c study, where the lesion appeared with an enhancing halo. Histopathologic reports for *in vivo* findings after LITT therapy in GBM have been recently reported in humans as well [35]. In this case, three distinct zones seemed to arise radially from the treatment site: a central necrotic area, a rim of granulation tissue with macrophage infiltration, and a more normal surrounding area with viable tumor cells. These findings are similar to what we experienced, with a central area of damage which becomes progressively more normal through a transition zone.

Lesion size in tcMRgFUS has been shown in the literature to increase significantly in size within 24 h of operation. The lesion size then shrinks chronically as gliogenic responses mount [36]. Of note, the time of exposure for NBTU varies greatly from that of tcMRgFUS (2–3 min compared to 17–240 min, respectively, to achieve similar lesion volumes) [37,38]. It has also been reported that the temperature required for lesioning was unable to be reached in 10% of cases [39]. In a swine study directly comparing the histologic and radiologic effects of FUS, RFA, and SRS, energy transfer in tcMRgFUS through the intact skull was ineffective and so a craniectomy/FUS model was adopted. Lesions were initially not apparent, however by 48 h post-ablation there were three characteristic zones: an inner zone of necrosis (hypointense), surrounded by a perilesional zone (markedly hyperintense) and a zone of surrounding vasogenic edema (diffusely hyperintense). The perilesional edema subsided within 10 days, and the lesion itself was difficult to appreciate by the 3-month MRI [40]. In this case, the histologic findings revealed discrete acute infarctions with central ischemic necrosis surrounded my edema, vacuolation of the neuropil, ischemic neurons, and axonal swelling. By 72 h there was an increase in macrophage infiltration.

The effects of SRS on native brain tissue have been observed through use of SRS in functional neurosurgical procedures (thalamotomy, pallidotomy, subthalamotomy). In these cases, lesions could be observed on the first post-operative MRI 3 months post-treatment as well-circumscribed contrast enhancing lesions with central hypointensity and surrounding fluid-attenuated-inversion-recovery (FLAIR) change [41]. The lesions remained visible on contrast enhanced MRI 1–2 years post-treatment, after which time the contrast enhancement regresses. A review of the MRI changes following SRS to 500 metastatic lesions between 2006 and 2009 demonstrated stable or reduced lesion size during the first 36 months. In one third of cases, there was a transient increase in lesion size (now colloquially termed pseudoprogression) typically between 3 and 6 months post-treatment with a peak volume around 15 months [42]. Thus, SRS has been associated with neurotoxicity and radiation necrosis, especially in the setting of primary brain cancer [43]. This can often be difficult to differentiate between true tumor growth and progression, and occasionally requires additional neurosurgical intervention to alleviate the mass effect [44]. A large multi-center study of SRS and fractionated SRS (fSRS) noted that the risk of post-treatment radionecrosis is proportional to dose and volume of treatment [45]. Histologically this appears as eosinophilic necrosis and gliosis with atypia as well as fibrinoid vascular necrosis and dystrophic calcification and surrounding clusters of macrophages [46].

### Use of FEM to predict ablation zones

The ability to comprehensively and reproducibly predict ablation zones based on intraoperative and post-operative imaging techniques is imperative for the implementation of thermal ablative techniques including MRgRA NBTU [47,48]. Continuous volumetric MRTI allows for the real-time thermal feedback necessary to perform successful NBTU, thereby limiting injury to adjacent, non-targeted regions. Similarly, FEM use a series of differential equations along with predetermined, tissue-specific acoustic absorption coefficients to simulate thermal dose distributions throughout the procedure and have been shown to correlate with MRTI mapping [19,49]. Here we have shown that our 3D MRTI accurately replicates FEM data. Both FEM and MRTI allow for a greater ability to noninvasively predict ablation, thereby reducing collateral damage to nearby structures [19]. MR and histological ablation zones at four weeks correlate but are greatly reduced in size in comparison to MRTI at the time of surgery. The strong histologic correlation to MRTI in the acute setting, which is confirmed by existing literature [17,50], supports the notion that the decreased size is due to the healing process which happens over time, rather than inaccurate imaging.

Extent of thermal ablation in brain tumors has been shown to correlate with progression free survival [51,52]. NBTU offers a potential advantage over existing therapies in that it can provide directional heating patterns (e.g., 180°, 360°). By utilizing directional probes as well as computernumerically-controlled (CNC) ablation, it is possible to create a large variety of shapes to match complex lesions [19].

### Use of CEM to monitor ablation zones

Stereotactic radiosurgery (SRS) has specific thresholds (isodose lines) to represent cumulative radiation, which must be respected when planning a treatment [53]. LITT and tcMRgFUS have adopted these strategies with isotherm lines. Because many different combinations of time and temperature can induce cellular death, CEM43 was established in an attempt to standardize the cumulative heat exposure introduced during a hyperthermic procedure [54]. CEM43 has allowed for the demonstration of notable thermal dose-effect associations, and thus continues to be widely used. In LITT, CEM43 isotherm lines (<2 min) are monitored in real time to avoid damage to critical structures [55]. In one study of tcMRgFUS-induced thalamotomy, authors correlated MRTI CEM43 thermal dose maps with post-procedure MRI images acquired 1-day post-treatment [31]. They noted best correlations for T2-weighted and T1-weighted at the 100 and 200 CEM43 isotherms.

In our experiments, the 360° probes induced smaller lesion volumes as compared to 180° by histology; in both cases this may have been due to adjacent heat sinks. For example, in one experiment heat was noted to have dissipated along an adjacent sulcus into the subarachnoid space, and in another the probe was directly adjacent to the ventricle and heat was noted to disperse into the ventricle. Using directional probes may provide a solution to this as it allows the user to preferentially choose an optimal target while avoiding undesirable areas (heat sinks, blood vessels, critical neurologic structures). Ultimately, using CEM data and histological validation, we predict the impact of these ablations both with 360° and 180° probes on normal brain parenchyma. We show that transition zones are at 345 microns with both probes. By carefully choosing applicator orientation, time of insonation, acoustic power supply, probe type (180°, 360°), ablation volumes can be tailored for lesions with complex shapes and sizes. At the moment, we are able to view the MRTI CEM thermal dose maps in real time as a way of guiding our ablation, and more specifically to limit off target heating. Ultimately the system will include drawing thresholds on the pre-operative volumetric MRTI images to guide an automatic termination to the treatment session (and likely also real-time manipulation of the robot for lesion-shaping). We will continue to develop and refine our techniques and also investigate the correlation with acute and subacute swine (who survive for 0–3 days post-ablation).

### Use of a large animal model

In this work, we first demonstrate a notable improvement over our previous survival swine study [50]. The use of a large animal model which we have employed in this study is useful as it allows technologies that are used in the clinic to be tested while affording the opportunity to have a pathological gold standard to compare with MR imaging. Direct correlation of MRTI thermometry with histology in postmortem tissue sections is only possible in animal models, emphasizing the need for rigorous pre-clinical studies such as described here to determine the appropriate thermal dosing parameters and distribution thermal interventions [17,50]. The effects of lesioning in normal brain tissue surrounding ablations has not been well defined *in vivo* for NBTU. We have previously demonstrated good correlation between the volumetric MRTI and acute tissue damage as demonstrated using 2,3,5-Triphenyltetrazolium chloride (TTC) staining [17]. To better examine this transition zone, we will employ acute animals in the future.

### Limitations

Limitations to this study include the limited number of swine, which limits statistical power. Further, the ablations were performed in healthy brain tissue, limiting the generalizability of the findings to pathologic tissue. How these findings will apply to brain tumors of varying type and tissue consistencies will need to be explored in the future. In these survival swine, the brain tissue outside the transition zone remained healthy one month after surgery; however, to better define and correlate FEM, CEM43, and histology, additional studies should be performed employing these techniques in acute and subacute animals. Additionally, while the ablation parameters in this study were pre-determined, the ultimate goal is for using real-time MRTI thermal dose maps to guide the ablation in real time, and in particular being able to denote zones which should receive lower doses and for automatic termination of the ablation or real-time manipulation of the robotic assistant.

These studies were performed in a young animal model, which may limit the generalizability to an adult model from an immunologic/inflammatory standpoint. A young animal model was chosen in part because of logistical considerations and to take advantage of institutional experience with farm swine of this age. An adult animal weighs hundreds of pounds and would pose several challenges in terms of transportation and recovery from anesthesia. A mini-swine model could be considered in future studies because their growth curve is not as steep as that of farm swine, but these can only be obtained select vendors in the U.S. and cost must be considered.

FEM are limited by their ability to define the precise zone of tissue ablation, especially in moving, asymmetric, heterogeneous tissues such as the brain because the thermal and photochemical properties of the tissues, as well as their perfusion volumes, are in a state of flux during the ablation procedure. In addition, there are innate limitations to the equations these models are based [56]. However the close correlation of MRTI and FEM, as well these MRTI sequences, to histological data in our prior work suggests adequate accuracy [17,19]. The final histologic volumes did appear significantly smaller compared to the intraoperative MRTI results, most likely due to tissue changes, healing, and growth of the swine and discrepancies between MRTI processing versus histologic processing. Additionally, the brain-cutting and MRI imaging are not guaranteed to be perfectly co-planar making direct comparison of the conformal patterns limited; however, this is somewhat mitigated by using thin-sliced 3D MRI protocols.

## Conclusion

We characterized intraoperative MRTI thermal isodose contours of MRgRA delivery of NBTU with post-operative MRI images and analyzed histological tissue sections to assess the transition zone. Limited effects were seen in surrounding normal tissue and the potential for conformal ablation was shown with 180° and 360° probes. Future work will focus on acute and subacute animals with 60° and 90° probes so that MRTI, treatment, and MRI imaging can be done more concurrently.

## Figures and Tables

**Table 1.** Material properties of the brain tissue-modeled acoustic medium.

Acoustic medium material properties [17,20]							
Heat capacity (*C_t_*)	Density (*ρ*)	Thermal conductivity (*k*)	Blood density (*ρb*)	Blood perfusion (*ω_b_*)	Speed of sound (*c*)	Attenuation (α_atten_)	Metabolic Heat (*Q_m_*)
3680 [J/kg/°C]	1035.5 [22]	0.565 [W/m/°C]	1050 [22]	0.0005 [22]	1551 [m/s]	31.95	25000 [W/m^3^]

**Table 2.** Ablation dose and duration parameters.

Ablation parameters and volumes									
	Side	Probe	Acoustic power (W)	Duration (s)	Absolute maximal temperature increase (°C)	Time to return to baseline (s)	MRTI volume (cm^3^)	Histology volumes (cm^3^)	Predicted volumes (cm^3^)

Swine 1	Left	360°, 7 mm	3	120	61	140	1	0.033	1.011
Swine 2	Right	180°, 7 mm	3	180	58	120	0.9	0.077	0.9375
Swine 3	Right	360°, 7 mm	4	120	59	130	1.2	0.030	1.2677
Swine 4	Left	180°, 7 mm	4	180	64	150	1.1	0.109	1.163

**Table 3.** Measured volume of hyperintensity (cm^3^) based on immediate, 2- week, and 4-week post-ablation scans.

Ablation regions over time			
Swine 1	Immediate	2 weeks	4 weeks

T1w	4.502	0.125	0.076
T1w+	NA	0.136	0.094
T2w	NA	0.193	ND
DWI	OA	0.318	ND
Swine 2	Immediate	2 weeks	4 weeks
T1w	2.782	0.244	ND
T1w+	NA	0.561	0.122
T2w	NA	0.305	ND
DWI	OA	0.079	ND
Swine 3	Immediate	2 weeks	4 weeks
T1w	4.555	0.043	ND
T1w+	NA	0.127	ND
T2w	NA	ND	ND
DWI	OA	0.171	ND
Swine 4	Immediate	2 weeks	4 weeks
T1w	4.946	0.105	ND
T1w+	NA	0.505	ND
T2w	NA	0.608	0.092
DWI	OA	0.526	ND

NA: MRI series was not acquired; OA: the ablation region was obscured by artifact; ND: the ablation lesion was not detectable.

## Data Availability

Raw data were generated at Albany Medical Center. Derived data supporting the findings of this study are available from the corresponding author J.P. on request.
